# Supplementary material for: The impact of chronic comorbidities at the time of breast cancer diagnosis on quality of life, and emotional health following treatment in Canada
Source: PLoS One. 2021 Aug 26;16(8):e0256536. doi: 10.1371/journal.pone.0256536 (PMC8389459; doi:10.1371/journal.pone.0256536)
Supplement: S1 Table — (DOCX) [file pone.0256536.s002.docx]

**S1 Table. Coding details of all variables included in Table 1, and corresponding questions, response options, and variable codes from the Canadian Partnership Against Cancer (CPAC) Transitions Study Survey**^a^**.**

| **Variable in Current Study** | **Corresponding Transitions Study Survey Questions** | **Variable Type and Categories**^b,c^ | **Transitions Study Survey Response Options** |
| --- | --- | --- | --- |
| Age  (covariate) | **Q3.** How old are you? | 7-category ordinal variable:   - 30-34 - 35-44 - 45-54 - 55-64 - 65-74 - 75-84 - ≥85 years | - Under 18 - 18 to 24 - 25 to 29 - 30 to 34 - 35 to 44 - 45 to 54 - 55 to 64 - 65 to 74 - 75 to 84 - 85 or over - Prefer not to answer |
| Marital Status | **Q4.** Are you currently…? | 3-category nominal variable:   - Single (REF) - Married or Partnered - Divorced or Separated or Widowed | - Single (never married) - Married - Partnered (living with someone) - Separated - Divorced - Widowed - Prefer not to answer |
| Household Size | **Q5.** Including yourself, how many people live in your household? | 5-category ordinal variable:   - 1 (I live alone) - 2 - 3 - 4 - 5 (5 or more) | - I live alone - 2 - 3 - 4 - 5 or more |
| Quality of Life  (outcome) | **Q8.** How would you describe your overall quality of life today? | 4-category ordinal variable:   - Very good - Good - Fair - Poor or Very poor | - Very good - Good - Fair - Poor - Very poor |
| Emotional Health  (outcome) | **Q9.** In general, would you say your emotional health is... | 4-category ordinal variable:   - Very good - Good - Fair - Poor or Very poor | - Very good - Good - Fair - Poor - Very poor |
| Number of Comorbid Conditions  (exposure) | **Q10.** Which, if any, of the following chronic conditions did you have before you were  diagnosed with cancer?  If you’ve been diagnosed with more than one type of cancer, please think about the  chronic conditions you had prior to your most recent cancer diagnosis.  Please X all boxes that apply to you | Sum of the six most prevalent chronic conditions (arthritis, cardiovascular disease, diabetes, osteoporosis, respiratory disease, mental health issues).  7-category ordinal variable:   - Zero - One - Two - Three - Four - Five - Six | - Arthritis, osteoarthritis, or other rheumatic disease - Cardiovascular or heart condition; hypertension or high blood pressure - Chronic kidney disease - Diabetes - Osteoporosis - Respiratory diseases (such as asthma or COPD - chronic obstructive pulmonary disease) - Mental health issues (such as depression or anxiety) - Other chronic condition. Specify type: ______________ - No chronic conditions |
| Physician in Charge of Follow Up | **Q20.** Since completing your cancer treatment, which physician has been in charge of overseeing your follow-up cancer care? | Binary   - Family doctor/general practitioner/nurse practitioner or Your oncologist, hematologist, surgeon, or other cancer specialist, or Both (REF) - No one or Unsure | - Family doctor/general practitioner/nurse practitioner - Your oncologist, hematologist, surgeon, or other cancer specialist - Both - No one - Unsure |
| Education | **Q76.** What is your highest level of education? | 3-category nominal variable:   - High school diploma or less (REF) - Post-secondary degree (Some college or technical school/CEGEP, or College or technical school/CEGEP, or Some university, or University undergraduate degree) - University graduate degree | - Grade school or less - Some high school - Highschool diploma or certificate - Some college or technical school/CEGEP - College or technical school/CEGEP - Some university - University undergraduate degree (Bachelor’s) - University graduate degree (Master’s or PhD) - Prefer not to answer |
| Employment Status | **Q79.** Which **ONE** of the following best describes your current employment situation? | 3-category nominal variable:   - Employed (full time, part time, on vacation or paid leave) (REF) - Unemployed or Homemaker or Student, or On paid sick leave/disability due to cancer or other reason - Retired | - Working full-time - Working part-time - On vacation or paid leave - On paid sick leave/disability leave due to cancer - On paid sick leave/disability leave due to other reason - I am a homemaker/stay-at-home parent - I am a full-time student - I am retired - I am currently unemployed - Prefer not to answer |
| Income^d^ | **Q80.** What is your total annual household income before taxes?  *If you are a student and your health care needs are financially supported by your parents,*  *please indicate your parents’ total annual household income before taxes* |  | - Less than $25,000 - $25,000 to less than $50,000 - $50,000 to less than $75,000 - $75,000 to less than $125,000 - $125,000 or more - Prefer not to answer |

Abbreviations: REF: reference category for ordinal variables.

^a^Transitions study survey data, the questionnaire, and codebooks, are available through the Canadian Partnership Against Cancer (CPAC), at the following link: <https://www.systemperformance.ca/transitions-study/transition-study-questions/>

^b^Variable categories are listed as included in the models (see Table 2 and Table 3 in the manuscript). The reference category for nominal variables is indicated as ‘(REF)’.

^c^Income was not included in any of the models, however the distribution of income was provided in Table 1. The income distribution includes the entire sample (n=3372) regardless of employment status (employed, unemployed, homemaker, student, on paid sick leave, retired).
