# Supplementary material for: The impact of chronic comorbidities at the time of breast cancer diagnosis on quality of life, and emotional health following treatment in Canada
Source: PLoS One. 2021 Aug 26;16(8):e0256536. doi: 10.1371/journal.pone.0256536 (PMC8389459; doi:10.1371/journal.pone.0256536)
Supplement: S2 Table — (DOCX) [file pone.0256536.s003.docx]

**S2 Table. Unadjusted estimates of the relationship between the number of chronic conditions at breast cancer diagnosis, and the study outcomes: (i) quality of life, and (ii) emotional health, among breast cancer survivors in the Transitions Study.**

| **Outcome** | **N (%)** | **Unadjusted OR (95% CI)** |
| --- | --- | --- |
| **Quality of life** |  |  |
| Very Good | 1349 (40.0) | REF |
| Good | 1486 (44.1) | 1.26 (1.16, 1.36) |
| Fair | 472 (14.0) | 1.86 (1.68, 2.06) |
| Poor/ Very Poor | 65 (1.9) | 2.43 (1.99, 2.98) |
| **Emotional health** |  |  |
| Very Good | 965 (28.6) | REF |
| Good | 1668 (49.5) | 1.17 (1.07, 1.27) |
| Fair | 615 (18.2) | 1.52 (1.38, 1.68) |
| Poor/ Very Poor | 124 (3.7) | 2.06 (1.76, 2.42) |

Abbreviations: N=number, OR=odds ratio, REF=reference category.
